# Cost-effectiveness of a clinical medication review in vulnerable older patients at hospital discharge, a randomized controlled trial

**DOI:** 10.1007/s11096-019-00825-3

**Published:** 2019-06-17

**Authors:** Amber A. W. A. van der Heijden, Martine C. de Bruijne, Giel Nijpels, Jacqueline G. Hugtenburg

**Affiliations:** 1 Department of General Practice and the Amsterdam Public Health Research Institute, Amsterdam University Medical Center, VUmc Location, Amsterdam, The Netherlands; 2 Department of Public and Occupational Health, Amsterdam Public Health Research Institute, Amsterdam University Medical Center, VUmc Location, Amsterdam, The Netherlands; 3 Department of Clinical Pharmacology and Pharmacy and the Amsterdam Public Health Research Institute, Amsterdam University Medical Center, VUmc Location, De Boelelaan 1117, 1081HV Amsterdam, The Netherlands

**Keywords:** Cost-effectiveness, Drug-related problems, Health care utilization, Hospital discharge, Medication review, The Netherlands

## Abstract

*Background* Drug-related problems (DRP) following hospital discharge may cause morbidity, mortality and hospital re-admissions. It is unclear whether a clinical medication review (CMR) and counseling at discharge is a cost-effective method to reduce DRP. *Objective* To assess the effect of a CMR on health care utilization and to investigate whether CMR is a cost-effective method to reduce DRP in older polypharmacy patients discharged from hospital. *Setting* 24 community pharmacies in the Netherlands. *Method* A cluster-randomized controlled trial with an economic evaluation. Community pharmacies were randomized to those providing a CMR, counseling and follow-up at discharge and those providing usual care. *Main outcome measures* Change in the number of DRP after 1 year of follow-up and costs of health care utilization during follow-up. In 216 patients the use of health care was prospectively assessed. Missing data on effects and costs were imputed using multiple imputation techniques. Bootstrapping techniques were used to estimate the uncertainty around the differences in costs and incremental cost-effectiveness ratios. *Results* CMR resulted in a small reduction of DRP. The proportion of patients readmitted to the hospital during 6 months of follow-up was significantly higher in the intervention group than in the control group (46.4 vs. 20.9%; *p* < 0.05). Health care costs were higher in the intervention group, although not statistically significant. The costs of reducing one DRP by a CMR amounted to €8270. *Conclusion* A CMR in vulnerable older patients at hospital discharge led to a small reduction in DRP. Because of a significantly higher use of health care and higher number of re-hospitalisations post CMR, the present study data indicate that performing the intervention in this patient population is not cost-effective.

## Impacts on practice

- Comprehensive transitional care programmes for vulnerable older patients at hospital discharge, partly based on patient-reported data, appear to be effective.
- The efficiency of comprehensive transitional care programs around medication review needs to be improved in order to achieve more tangible results (e.g. less re-hospitalisations) and become cost-effective.

## Introduction

Since the publication of the report ‘To Err Is Human’ by the US Institute of Medicine in 1999 concerning the frequent occurrence of DRP, awareness of medication safety has strongly increased [1]. DRP may cause morbidity, mortality and re-admission, particularly in elderly patients with chronic disorders who are discharged from hospital [2–4]. DRP are not only a burden to patients and their relatives but also to society as they may incur high costs [5–7]. In 2008 the Hospital Admissions Related to Medication (HARM) study showed that 5.6% of the unplanned hospital admissions in the Netherlands were medication-related, of which half might have been prevented [8]. As the result, substantial but still insufficient [9] effort has been made to implement programs to reduce the occurrence of DRP and related hospital admissions.

The involvement of pharmacists in the care of older patients with polypharmacy is one of the most commonly described measures to optimize prescribing and reduce DRP [3, 10]. In the UK the National Service Framework for Older People recommends to regularly conduct medication reviews accompanied by paid medicine management services [11]. Recent systematic reviews on the effects of transitional care interventions including CMR on clinical outcomes, health care utilization and costs showed that the number of DRP may be reduced but that the effect on health care utilization and hospital (re-) admissions in particular, is ambiguous [12–14]. In addition, the number of studies investigating the cost-effectiveness of a CMR is limited and their results are also heterogeneous [11, 14, 15]. However, it is not unlikely that the benefit of a CMR will be especially applicable to patients with a high risk of DRP, such as older patients using a variety of drugs for the treatment of chronic disorders and those with one or more chronic disorders discharged from hospital.

## Aim of the study

The aim of the present study was to assess the effect of a CMR on health care utilization and to investigate whether CMR is a cost-effective method to reduce DRP in older patients with chronic disorders using a combination of drugs who are discharged from hospital.

## Ethics approval

The study was approved by the Medical Ethics Committee of the VU University Medical Center Amsterdam. All patients provided informed consent at the start of the study. The Dutch trial register number is NTR-1194.

## Method

### Study design and population

**Fig. 1:**
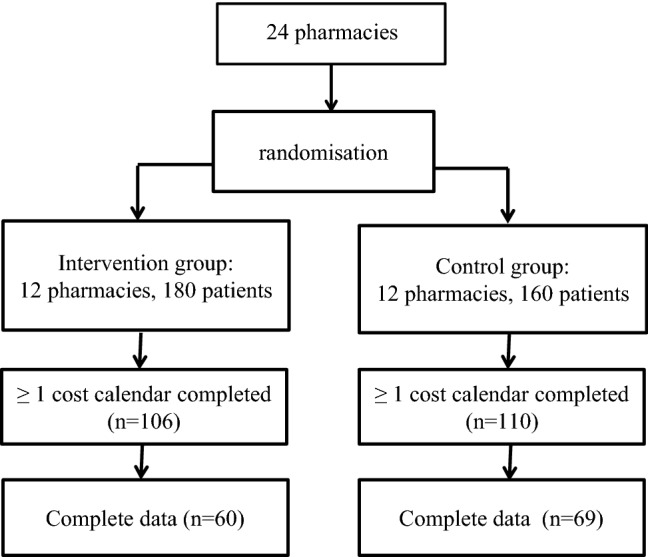
Overview of pharmacy randomisation, number of patients included in the study and number of patients with available cost data

The present study (IBOM-2) investigated the effect of a CMR on the occurrence of DRP in older patients with a chronic disorder using five or more prescription-only drugs discharged from hospital [16]. Twenty-four community pharmacies in the Amsterdam area participated in the study. They were randomized into pharmacies conducting the CMR plus usual care and those providing usual care only (Fig. 1). Each pharmacy was instructed to include between 15 and 20 patients registered at the pharmacy. Following receipt of the discharge prescription/medication list from the hospital (as is usual in the Amsterdam area) the pharmacist determined whether a patient was eligible to participate in the study on the basis of the patients’ age and medication record data. Patients 60 years or older using at least five prescribed drugs were eligible to participate. Patients discharged from psychiatric or oncology departments were excluded as well as patients discharged to a nursing home. Patients needed to understand the Dutch language. Those who consented were included in the study [16].

### Intervention

The design of the study has previously been described in detail [16]. Pharmacists were instructed to conduct a CMR (after having received the discharge prescription/medication list from the hospital and subsequent verification [reconciliation]) including a medication analysis, treatment analysis, patient interview and counseling. DRP were identified by using the Amsterdam CMR tool, a comprehensive checklist of 124 DRP divided by 20 sections according to physiological systems and diseases and including a structured interview script for a patient interview [17]. DRP were categorized using the Pharmaceutical Care Network Europe DRP-score form [18]. Medication records kept in the electronic pharmacy administration and information systems (PAIS) of the participating pharmacies listing all drugs prescribed and dispensed during the 6 months preceding the date of discharge (including those prescribed by the hospital and used at discharge) were printed. PAIS were also used for the identification of possible drug interactions. The semi-structured patient interview script was used to identify DRP experienced by patients like ineffectiveness of treatment, side effects, and fear of side effects and non-adherence [16, 19]. The patients’ GP and, if applicable, medical specialists were contacted by the pharmacies for information about the chronic diseases of the patients, indications for drugs and results of laboratory tests. For the medication and treatment analysis, all prescribed drugs dispensed during the last half year preceding the date of inclusion were evaluated with respect to drug choice, dosing, drug interactions and (potential) DRP [16].

Causes of DRP were assessed and communicated with the patient and GP which could result in an adaptation of the drug regimen. Patients in the intervention group were informed about the use, effects and side effects of their medication. At baseline, they were motivated by a pharmacy staff (pharmacist or trained technician) with the help of a structured interview protocol [19] to sustain or improve their drug adherence. Home supplies of drugs were checked and synchronized at each visit. All patients were handed a written outline of their drug regimen. Cancelled and redundant drugs were taken in. After 1 year the medication used by the patient was assessed again on potential DRP [16].

### Control group and usual care

Control pharmacies provided usual care according to the Dutch Pharmacy Standard [16]. PAIS routinely check prescriptions for drug interactions and contraindications. Data on medication was retrieved from the PAIS. Eventually, GPs were contacted for information on the patients’ chronic disease. Control patients were visited once by a researcher and interviewed about DRP experienced after discharge from hospital. After 1 year the medication used by the patient was assessed again on (potential) DRP [16].

### Clinical outcome measure

The clinical outcome measure was the difference in the number of DRP between intervention and control group after 1 year of follow-up. DRP were blindly assessed by two independent clinical pharmacologists [16].

### Cost measures

Formal and informal health care utilization was assessed from a societal perspective using monthly costs calendars in which the use of health care was prospectively registered by the patient. The following resources were included in the calendars: GP (home) visits, visits to a medical specialist, use of physical therapy, home care and number of days of re-hospitalisation. Use of informal care such as help from family, friends and neighbours was also included. Cost calendars were provided to the patients at the start of the study. Patients were asked to fill out the calendar each time they used health care and to return the calendar to the researchers at the end of each month. Patients who did not return the calendars were contacted by the researchers. Costs of resource use were calculated using Dutch unit prices (Table 1) [20].

**Table 1:** Baseline characteristics of the study population (patients having completed at least 1 cost calendar) according to randomization

	Patients control group	Patients intervention group	*P* value
	(n = 110)	(n = 106)	
Age (years)	73.9 (8.3)	75.5 (9.2)	0.19
Men (%)	43.6	51.9	0.23
Educational level (%)			
Low	31.2	33.3	0.70
Medium	51.6	45.7	
High	17.2	21.0	
Dutch nationality (%)	94.8	92.0	0.84
Number of medications used	8.4 (3.0)	8.9 (2.7)	0.225
Number of diseases	3.3 (1.7)	2.7 (1.4)	0.01
Number of drug-related problems	1.6 (1.5)	1.6 (1.3)	0.94

Data are presented as mean (SD) or proportions

### Additional measures

Information on educational level and nationality of the patient was obtained using self-administered questionnaires. The number of medications used by the patient was determined during the medication analysis. The number of chronic diseases was assessed using GP medical record data of each patient [16, 19].

### Statistical analysis

In this vulnerable population part of the data was missing. Data of complete cases, i.e. patients who completed six subsequent calendars after hospital discharge, were used to investigate details on health care utilization. Data of patients with at least one calendar during the first 6 months after hospital discharge were multiply imputed. Missing data on effects and costs were imputed using multiple imputation by chained equations using predictive mean matching. Baseline variables related to missing data on costs and effects and baseline variables associated with costs and effects (age, sex, educational level, number of drugs, number of DRP, number of diseases and the available information on costs) were included in the multiple imputation model. Multiple imputation was performed in SPSS 20.0, and 10 complete data sets were generated. The results of these datasets were pooled using Rubin’s rules [21].

Baseline characteristics of the patients were presented as mean values (± standard deviation) or proportions according to randomization. Baseline similarity between the intervention and control group was studied using independent t-tests for continuous variables and Chi2 tests for categorical and dichotomous variables. To investigate change in the number of DRP during 1 year of follow-up between the intervention and control group, paired t-tests were performed and effect sizes were estimated.

Health care utilization during the first 6 months after hospital discharge was calculated and presented as proportions of patients that used the specific health care resource and mean (SD) number of visits per patient for the specific health care resource. Differences in health care utilization between patients allocated to the intervention or control groups were tested using Χ^2^ tests and Mann–Whitney test. Despite the skewed distribution of health care utilization and costs in our population, these measures were presented as means because this is the most informative measure from an economic perspective [22].

Because of the skewed distribution of cost data, bootstrapping methods (5000 replications) were used to estimate “approximate bootstrap confidence” (ABC) intervals around cost differences [23, 24]. Incremental cost-effectiveness ratios (ICERs) were calculated by dividing the difference in costs by the difference in effects between the intervention and the control group. The 95% confidence intervals around the mean difference in costs and the uncertainty around the ICERs were estimated using non-parametric bootstrapping with 5000 replicates. *P* values below 0.05 were considered as statistically significant. In a cost-effectiveness plane (CE plane), we plotted incremental costs (y axis) and effects (x axis) between the intervention and control group resulting in four quadrants. The uncertainty around the ICERs was depicted by plotting the bootstrapped cost-effect pairs on a CE plane. In a sensitivity analysis, the cost-effectiveness analysis was repeated using data of complete cases only. The cost-effectiveness analysis was performed in R Statistical Software (version 2.13.1). Other analysis was performed in SPSS version 20 for Windows.

## Results

Baseline characteristics of the study population are shown in Table 1. In patients of the intervention pharmacies the number of chronic diseases was lower as compared to patients of the control group (2.7 vs. 3.3). Other characteristics did not differ between the two groups. Of the 340 patients included in the trial and evaluated with respect to occurrence of DRP [19, 25], 35 patients (10.3%) died during the 1 year follow-up. The mortality rate did not significantly differ between the intervention (11.7%) and the control groups (8.8%). Overall, patients who died during follow-up were more likely to be male (13.9%) (*p* = 0.04). Other characteristics did not differ between patients who died during follow-up and those who survived.

Using data from patients who completed at least one cost calendar larger decreases in the number of DRP after 12 months of follow-up were seen in the intervention group than in the control group. The effect of the CMR on the number of DRP was small but statistically significant (− 0.2 [95% CI: − 0.4 to 0.0]) (Table 2). This result is essentially similar to that obtained by analyzing the data of all patients initially included (intervention group: N = 180 vs control group: N = 160). More detailed data on the DRP found in this population have been reported elsewhere [25].

**Table 2:** Pooled mean effects (SE) and differences in number of drug-related problems over 12 months of follow-up

	Number of drug-related problems			
	Baseline	12 months	Mean difference during follow-up	Mean effect difference
Patients control group (N = 110)	1.6 (0.1)	1.6 (0.1)	0.00 (− 0.1 to 0.2)	
Patients intervention group (N = 106)	1.6 (0.1)	1.4 (0.1)	− 0.2 (− 0.4 to -0.1)	− 0.2 (− 0.4 to 0.0)

At least one monthly cost calendar during the first 6 months after hospital discharge was completed by 216 patients (63.5%). In this vulnerable population, 123 (36.2%) patients completed the first six cost calendars with information on use of health care (complete cases) (Fig. 1). Patients with complete data were younger (− 2.2 years; *p* = 0.03), had less DRP at baseline (− 1.8; *p* < 0.001), used less medication (− 0.7; *p* = 0.04) and were more likely to be randomized to the control group as compared to patients without complete data.

During the first 6 months after hospital discharge patients in the intervention group more frequently visited their GP, medical specialist and physical therapist, although these differences were not statistically significant (Table 3). Within 6 months after baseline, significantly more patients in the intervention group (46.4%) were readmitted to hospital as compared to those in the control group (20.9%). The mean number of days of readmission was also higher in the intervention (7.0 days) than in the control group (3.4 days) (difference not statistically significant).

**Table 3:** Health care use in the first 6 months after discharge from hospital: analysis on cases with complete data on health care use, costs and effects

	Unit	Unit cost (€, 2009)	Patients control group		Patients intervention group	
			N = 67		N = 56	
			Patients using resource (%)	Mean (SD)	Patients using resource (%)	Mean (SD)
General practitioner	Visit	22	71.6	2.9 (4.3)	85.7	3.8 (3.3)
General practitioner	Home visit	44	47.8	1.2 (2.6)	57.1	1.4 (1.9)
Medical specialist	Visit	61	97.0	9.2 (9.6)	94.6	9.9 (9.6)
Physical therapist	Visit	25	40.3	9.8 (15.5)	53.6	6.7 (12.3)
Hospital readmission	Day	394^$^	20.9	3.4 (9.4)	46.4^a^	7.0 (17.3)
Home care	Hour	34	31.3	17.4 (37.5)	16.1^a^	7.0 (19.9)
Help by friends/family	Hour	9	14.9	16.2 (81.3)	23.2	113.1 (527.3)
Paid housekeeping	Hour	14	28.4	35.7 (99.2)	23.2	22.1 (49.8)
CMR^#^		70			100	

^a^Indicates a significant difference between the control and intervention group (*p* value < 0.05)

^$^Sum of the proportion of patients admitted to an academic hospital (0.16 * €522) and general hospital. (0.84 * €370)

^#^*CMR* clinical medication review

**Fig. 2:**
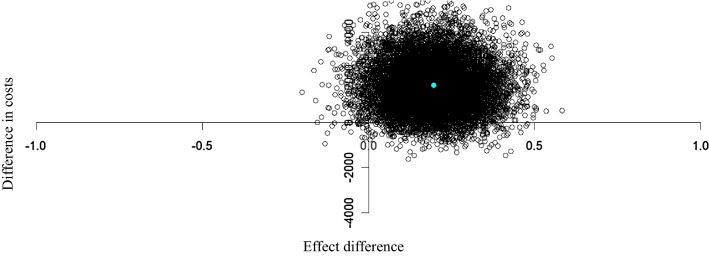
Cost-effectiveness plane for the difference between the intervention and control group in the difference of drug-related problems after 1 year of follow-up. An effect difference > 0 means that after 12 months of follow-up, the decrease in the number of drug-related problems was higher in the intervention group as compared to the control group

Costs of resource use in the intervention group were €1654 higher than in the control group (95% CI: − 520 to 3828), although not statistically significant (Table 4). These higher costs were mainly caused by the large number of patients with hospital readmissions in the intervention group. Figure 2 shows the cost-effectiveness planes for the difference in number of DRP determined by medication analyses over 12 months of follow-up. The majority of the cost-effect pairs was located in the northeast quadrant of the cost-effectiveness plane indicating that higher effects were accompanied by higher costs of the intervention compared to control. The costs of reducing one DRP by a CMR amounted to €8270.

**Table 4:** Mean differences in total costs (Euros) and effects (95% confidence intervals (CI)) between the intervention and the control group, incremental cost-effect ratios (ICERs), and cost-effectiveness (CE) plane distributions

Multiple imputed	Patients control group (N = 110)	Patients intervention group (N = 106)	∆ Costs (95% CI) (Euros)^a^	∆ Effects (95% CI) (difference in DRP)^b^	ICER for improvement in DRP	Distribution CE plane (%)			
	Mean total costs (se)	Mean total costs (se)				NE^c^	SE^d^	SW^e^	NW^f^
Model 1^g^	3796 (437)	5450 (1035)	1654 (− 520 to 3828)	− 0.19 (− 0.42 to 0.03)	8705	90	5	0	4
Model 2^h^	3796 (437)	5450 (1035)	1654 (− 520 to 3828)	− 0.20 (− 0.40 to 0.04)	8270	92	06	0	2
Complete cases	N = 67	N = 56							
Model 1^g^	3493 (588)	5335 (1595)	1842 (− 337 to 8100)	− 0.10 (− 0.42 to 0.22)	18,420	62	10	5	23
Model 2^h^	3493 (588)	5335 (1595)	1842 (− 337 to 8100)	− 0.06 (− 0.34 to 0.22)	30,700	56	9	3	32

^a^Costs include formal and informal costs

^b^*DRP* drug-related problems

^c^Refers to the northeast quadrant of the CE plane, which indicates that medication review is more effective and more costly than usual care

^d^Refers to the southeast quadrant of the CE plane, which indicates that medication review is more effective and less costly than usual care

^e^Refers to the southwest quadrant of the CE plane, which indicates that medication review is less effective and less costly than usual care

^f^Refers to the northwest quadrant of the CE plane, which indicates that CMR is less effective and more costly than usual care

^g^Unadjusted analysis

^h^Adjusted for baseline number of DRP

Complete case analysis showed the same trend as compared to analysis in the multiple imputed population. Of the cost-effect pairs, the majority was located in the northeast quadrant. A CMR at discharge was more costly and but only slightly more effective than usual care regarding the reduction of DRP (Table 4).

## Discussion

A CMR on the basis of both medication records and patient reported data, and including counseling sessions, for elderly patients with a chronic disease using multiple drugs at hospital discharge resulted in an overall reduction of DRP after 1 year of follow-up. The overall reduction was achieved by a slightly reduced number of DRP identified by medication analysis on the basis of PAIS data and a substantially reduced number of DRP identified with the patient interview. In the control group the numbers of both categories of DRP were slightly increased [25].

Recent study data show that it is important to identify and address patient-reported DRP, notably adverse effects affecting patients in their daily routines and well-being. They strongly contribute to therapeutic ineffectiveness largely as the result of suboptimal medication use and unintentional and intentional non-adherence the latter phenomenon predominantly resulting from adverse effects [16, 19, 26]. Therefore, efforts to obtain these data by means of interview or questionnaire should be an integral part of any CMR procedure [12, 16, 27, 28]. However, conducting CMR may not result in substantial reductions of the overall number of DRP found [12, 13, 29]. In the Netherlands this phenomenon among other things can be explained by the high level of usual care including the common use of advanced PAIS [30]. Over the last decades several CMR elements increasingly have become part of usual care including those highly relevant in the case of care transitions. Moreover, the vast majority of patients only use the services of a single community pharmacy. Clearly, differences in outcomes between advanced care, of which a CMR is still a part of, and usual care therefore will be smaller and more difficult to detect.

Due to a lack of data, mainly because relatively few patients returned these questionnaires (possibly as the result of the 1 year follow-up period being too long), the effect of the CMR on the patient’s quality of life could not be assessed. In this respect, in the literature there is hardly any evidence showing that (pharmaceutical) transitional care interventions have a clear positive effect on the quality of life. Particularly in the case that patient-reported information on adverse events is not used or the follow-up period is very long, it is possible that for patients there is no relationship between the reduced risk of medication-related events resulting from the intervention and their quality of life. However, as in the preceding IBOM-1 study comprising a medication review on the basis of PAIS data alone and patient counseling shortly after discharge [31], most patients were well satisfied with the intervention [19].

The effects of a CMR on patient outcomes have been the subject of several studies. Similar to the present study, results obtained in older patient populations generally show a positive effect including a reduction of the occurrence of DRP, improvement of medication appropriateness and a decrease in the number of prescriptions [13, 32–36]. However, trial results with respect to the effects of a CMR on health care utilization are inconclusive [11–15]. These studies focused on patients from a variety of older patient populations and settings, including residential homes, primary care patients, mental health problems or patients with diabetes, hypertension or depression. Studies in older patients discharged from hospital were not included. In those few studies that investigated the cost effectiveness of the intervention, results were ambiguous. In the HOMER trial the effect of a CMR led to an increase of the number of admissions by 30% and of GP home visits by 43% in the first 6 months after hospital discharge [37]. The increased number of GP visits in the intervention group was attributed to pharmacists’ efforts to increase the understanding of patients about their conditions and medication use which was considered to have increased patient awareness and help seeking behaviour [37].

In the present study the rate of hospital re-admissions of patients in the intervention group was also significantly higher. Overall costs for these patients were also higher than those of the control group. There is no clear explanation for these findings. In the context of the Dutch healthcare system with its easy access to primary care and managed access to hospital care it is rather unlikely that increased help-seeking behaviour would have contributed to the increased number of hospitalisations nor that they would have been unnecessary. Notably in view of the somewhat limited size of the study, we feel that the higher costs of care in the intervention group, particularly those brought about by hospital re-admissions, are most likely due to chance. On the other hand, counseling sessions focusing on the use, effects and side effects of the medication used indeed may have increased patient awareness which might have increased the number of GP visits. Nevertheless, in its present form a CMR at discharge is not cost-effective.

An important outcome of our study was the high number of patients that were not treated with the medication recommended by disease specific guidelines. The presence of these drugs in the medication of the highly vulnerable population of our study possibly might also have led to an increased use of health care and costs. It should be investigated whether interventions aimed at deprescribing of inappropriate medicines in this population of vulnerable older patients reduces the rate of rehospitalisation.

A limitation of our study is the considerable amount of missing data limiting the power of this study. Patients with missing data were older, lower educated, had more DRP at baseline, used more medication and were more likely to be randomized to the intervention group as compared to patients without complete data. However, we used multiple imputation techniques to impute missing data taking into account all available information related to the missing data. Despite these methods, the possibility exists that the results of this study were affected by the missing data. In the multi-imputed case analysis, the difference in DRP was slightly increased as compared to the complete case analysis, most likely as the result of a selective drop-out of patients caused by transfer to nursing homes and death of the most vulnerable patients. However, the small difference did not influence the conclusion of this study and cost-effectiveness analysis results using data after multiple imputation techniques were in line with the analysis on complete cases. A second limitation of this study is that no distinction was made between the type of DRP and the risk of harm associated with the specific DRP. Weighting of DRP might have affected the effect estimates but not our conclusions.

## Conclusion

In older patients using five or more chronic drugs discharged from hospital, the gain of a small reduction in DRP induced by conducting a CMR is offset by higher hospital readmissions, use of health care and costs in the period thereafter. In line with the results of a recent study on the cost-effectiveness of a transitional care program in the same region comprising several elements of the present intervention [38], the efforts and costs of these comprehensive interventions do not justify their widespread use. However, in view of the continuing presence of DRP in older patients at hospital discharge and their considerable consequences and costs [6, 8, 9, 15, 39], ways have to be found to address this persistent problem and prevent unnecessary harm but control intervention costs. Interventions clearly need to be standardized, simplified and adequately funded to ensure their full and complete application. Collaboration between health care providers (including nurses and pharmacy staff) also needs to be intensified. However, the introduction of measures such as the use of adequate (electronic) communication protocols at discharge, integration of deprescribing in CMR, thereby increasing awareness for the discontinuation of inappropriate medication, and more efficient means to obtain patient-reported information on symptoms should also be considered.
